# Attentional Differences as a Function of Rock Climbing Performance

**DOI:** 10.3389/fpsyg.2020.01550

**Published:** 2020-07-23

**Authors:** Inmaculada Garrido-Palomino, Simon Fryer, Dave Giles, Javier J. González-Rosa, Vanesa España-Romero

**Affiliations:** ^1^MOVE-IT Research Group, Department of Physical Education, Faculty of Education Sciences University of Cádiz, Cádiz, Spain; ^2^School of Sport and Exercise, University of Gloucestershire, Gloucester, United Kingdom; ^3^Lattice Training Ltd., Chesterfield, United Kingdom; ^4^Neuroimaging and Psychophysiology Group, Department of Psychology, University of Cádiz, Cádiz, Spain; ^5^Biomedical Research and Innovation Institute of Cádiz (INiBICA), Puerta del Mar University Hospital, Cádiz, Spain

**Keywords:** attention, climbing ability, physical condition, performance, on sight, red point, selective attention

## Abstract

The purpose of this study was to investigate the relationship between attention (using two different attention tasks) and self-reported climbing ability while considering potential confounding factors (sex, age, climbing experience, and cardiorespiratory fitness) in a group of experienced climbers. Accuracy of response (AC) and reaction time (RT) from two different attention tasks using the Vienna Test System, along with self-reported on-sight and red-point climbing ability, were assessed in 35 climbers. Linear regression revealed that climbers with the highest self-reported on-sight grade had better AC during the attention task. Linear regression models revealed, after controlling for potential confounders, that AC, measured using two attention tasks, was positively related to climbers’ highest self-reported on-sight climbing ability (β = 0.388; *p* = 0.031). No significant differences were found between AC and self-reported red-point climbing ability (β = 0.286; *p* = 0.064). No significant relationship was found between RT and climbing ability (β = −0.102 to 0.020; *p* = 0.064). In conclusion, higher-level rock climbers appear to have an enhanced attention, which is related to on-sight lead climbing style, and thus, it may be an important component of climbing performance. Coaches should consider incorporating techniques to train attention based on on-sight climbing style in climbers.

## Introduction

Attention is a central feature of all perceptual and cognitive functioning (Chun et al., 2011), which allows for the selection and processing of information (Kahneman, 1973; Lavie, 2005). Attention is composed of three distinct networks which are responsible for controlling different attentional functions; they are orienting, alerting, and executive control (Posner, 2017). Orienting is primarily responsible for the ability to prioritize sensory input by selecting a modality. Alerting serves to produce and maintain optimal levels of arousal and performance, a necessary prerequisite for other attention functions (Peterson and Posner, 2012). Finally, executive control is responsible for directing attention to relevant and useful information, away from irrelevant information, and also for inhibiting extraneous stimuli (Roderer et al., 2012). Collectively, these systems can be overloaded when individuals attempt to multitask and divide their attentional capacity between selecting information and deciding on action strategies. Consequently, it is unsurprising that in sports, attention appears to be related to sport practice (Kioumourtzoglou et al., 1998; Williams and Davids, 1998; Heppe et al., 2016; Sanchez-Lopez et al., 2016; Qiu et al., 2018; Meng et al., 2019). This has been shown to be the case in sports such as martial arts (Sanchez-Lopez et al., 2016), basketball (Qiu et al., 2018), soccer (Heppe et al., 2016), and volleyball (Kioumourtzoglou et al., 1998). However, the differences in attention responses seem to depend on the influence of a variety of information processing demands associated with each sport’s modality (Singer, 2000; Voss et al., 2010).

Specifically, in climbing, several authors have studied attention and climbing performance (Bourdin et al., 1998; Nieuwenhuys et al., 2008; Green and Helton, 2011; Young, 2011; Green et al., 2014). It has been suggested that climbing performance could be associated with attentional control (Young, 2011), which is associated with postural control and climbing route difficulty (Bourdin et al., 1998). Young (2011) demonstrated that while ascending, climbers who were distracted (via cognitive interference) by a task that necessitated a heightened degree of attention performed significantly worse (i.e., in terms of increased climbing time) than non-distracted climbers. Similarly, the effect of attentional interference on climbing performance was demonstrated by Green and Helton (2011). The authors suggested that when climbers use their attentional resources in a task other than climbing (i.e., a memory task), climbing efficiency and distance ascended decreased. It has also been shown that attentional demands increase with the difficulty of a climbing task, which further affects climbing efficiency (Bourdin et al., 1998). Despite the literature describing the influence of attention on different aspects of climbing performance, to date, data investigating the relationship between attention and climbing performance remain limited. Given the psychophysiological demands of rock climbing ability (Giles et al., 2014) which encompass physical and tactical elements combined with complex psychological traits such as a high self-confidence and low trait anxiety (Aras and Ewert, 2016), this lack of research seems unusual.

As rock climbing ability has previously been associated with a high cardiorespiratory fitness (CRF) (Aras and Ewert, 2016) and this is known to be related to attention (Colcombe and Kramer, 2003; Kramer and Colcombe, 2018), it may be that CRF also has some influence on climbers’ attentional performance. Greater CRF appears to be associated with a better cognitive function, which is related to an increased ability of the heart to deliver oxygenated blood to cerebral structures (Colcombe and Kramer, 2003), cerebral blood flow (Brown et al., 2010), and a brain-derived neurotrophic factor (Vaynman et al., 2003). Luque-Casado et al. (2016) suggested that there is likely to be a relationship between CRF and sustained attention in sports performance. Those authors observed that cyclists and triathletes with higher CRF had shorter reaction times (RTs) than those with a lower CRF during a sustained attention task. Further, it has also been proposed that nonathletes with a high CRF may have a better ability to allocate attentional resources over time compared to nonathletes with a low CRF during a sustained attention task (Ciria et al., 2017). Further, Sanabria et al. (2019) speculated that the relationship between CRF and sustained attention may be dependent on the type of sport being conducted. Given that rock climbing has been shown to independently have both a high CRF (Fryer et al., 2017) and a high attention demand (Bourdin et al., 1998; Green and Helton, 2011), CRF may be a physiological mediator that could at least in part explain on-sight and red-point ability. To our knowledge, the relationship between ability level (on-sight and red-point), CRF, and attention in rock climbers has not yet been studied. Therefore, the main purpose of the present study was to investigate the relationship between attention (using two different attention tasks) and self-reported climbing ability, taking into account potential confounding factors (sex, age, climbing experience, and CRF) in a group of experienced climbers.

## Materials and Methods

### Participants

Thirty-five sports climbers (10 women), mean age 34.7 ± 6.2 years, volunteered to take part in the study. All participants were healthy, were nonsmokers, and were not taking any vascular acting medication. Participants were asked not to consume food for 4 h prior to testing and to avoid caffeine and exercise for a minimum of 12 h. All testing sessions were conducted in the same week, in an environmentally controlled exercise laboratory. Participants read and signed the informed consent prior to participation in the study. The study protocol was approved by the Institutional Review Committee for Research Involving Human Subjects prior to recruitment; data collection was performed in accordance with the ethical standards set by the journal and the Declaration of Helsinki. Data from this study come from the High-Performance International Rock-Climbing Research Group (C-HIPPER).

### Procedure, Apparatus, and Materials

Participants visited the laboratory once. During the visit, each participant completed forms for the determination of informed consent, health history, and demographic data. Detailed information on climbing experience (years), frequency (days per week), and self-reported rock climbing ability were recorded. Two attention tasks (Signal Detection and Determination Tasks) were administered (counterbalanced) with a 30-min break between each, using a laptop (15 in., 1,366 × 768 color screen) running the Vienna Test System software version 26.04 (Schuhfried, Austria). In addition, participants completed an incremental treadmill cardiorespiratory exercise test to determine CRF.

#### Self-Reported Climbing Ability

Rock climbing ability is most commonly expressed in terms of the best ascent of a route within the last 6–12 months. Routes are ascended as either on-sight (no prior knowledge or visual route inspection requiring a screening to find new holds) or red-point (pre-practiced where the athlete remembers the location of each hold and the movement required). Climbing ability was reported as the best grade achieved 6 months prior to the study. Self-report has been used for on-sight and red-point performance extensively within the literature (Baláš et al., 2017; Fryer et al., 2017; Zarattini et al., 2018). It has been shown to be a valid assessment of on-sight ability level (Draper et al., 2011a). Climbers had a best 6-month on-sight ability ranging from 6a+ to 8a+ and from 6b to 8b+ for the best 6-month red-point ability based on the French grading system. In brief, this system is based on a scale of integers ranging from 4 (very easy) upward to 9 (very difficult) with letter subdivisions of a, a+, b, b+, c, and c+ from 6a to upward. In accordance with the Position Statement by the International Rock Climbing Research Association (IRCRA; Draper et al., 2016), performance grades were converted from French Sport to specific numerical values (IRCRA grades) to enable calculations and statistical analyses. The IRCRA scale ranges between 1 (very easy) and 32 (very difficult), for reporting climber ability (Draper et al., 2011a, 2016). Climbers had a best 6-month on-sight ability ranging from 12 to 24 and red-point from 13 to 26 based on the IRCRA scale (see Table 1).

**TABLE 1:** Mean (SD) of the anthropometric, demographic, physical fitness, and performance data in the care tasks of the participants of this study.

	**All (*n* = 35)**	**Male (*n* = 25)**	**Female (*n* = 10)**
Age (years)	34.7 (6.2)	33.5 (6.5)	37.9 (4.2)*
Mass (kg)	64.5 (8.6)	68.3 (6.7)	55.2 (5)^+^
Height (cm)	171.5 (8.0)	173.7 (8.0)	166 (4.9)^+^
Experience (years)	11.1 (7.0)	11.5 (7.6)	10.1 (5.7)
**Self-reported climbing ability**			
Best 6-month on-sight grade (French)	7a (3.0)	7a^+^ (2.9)	6b^+^ (1.6)*
Best 6-month red-point grade (French)	7a+ (3.6)	7b (3.7)	6c^+^ (1.6)*
**Treadmill measures**			
Cardiorespiratory fitness (ml ⋅ kg ⋅ min^–1^)	48.6 (5.3)	50.7 (4.9)	45.1 (4.6)*
Heart rate (bpm)	186.4 (10.5)	188.0 (10.9)	182.6 (8.9)

*p < 0.05; ^+^*p* < 0.001.

### Attention Tasks

#### Signal Detection Task

**FIGURE 1:**
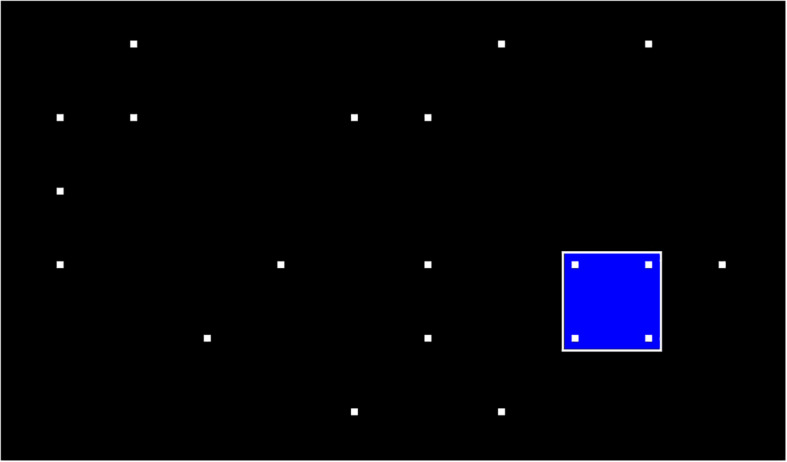
Signal detection task from vienna test system.

The Signal Detection Task (SIGNAL, 26.04 versions, Vienna Test System) was used to evaluate the accuracy of participants’ response (AC) to a visual scanning and selective attention (Chong and Ong, 2015). This task was characterized by the presentation of an infrequent and unexpected target among frequent nontarget stimuli (distractors) for a relatively long period of time, requiring participants to be precise in order to detect the objective stimulus between the distractors. Specifically, during the SIGNAL task, white dots pseudorandomly disappear and appear on a black background. Participants were instructed to press the indicated key with the index finger of their dominant hand each time they detected a programmed stimulus constellation, created by four points that formed a square (see Figure 1 for an illustration of technical terms). Climbers used headphones while performing the tasks to reduce distraction because of background noise.

The SIGNAL task had a total duration of 840 s (including the instruction and practice phases). The main practice phase had 1,000 point changes with a total of 60 stimulus constellations, this task being only trial. The number of correct responses on time as a measure of AC in the execution of the task was collected in the percentage for the final analysis.

#### Determination Task

**FIGURE 2:**
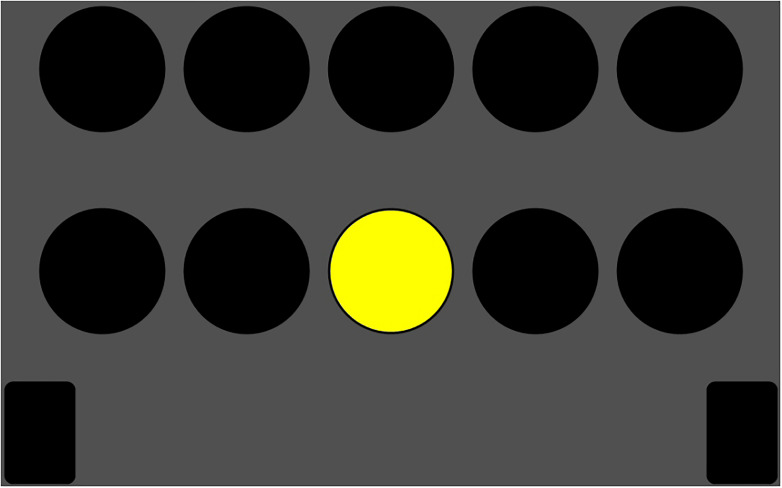
Detection task (modified version) from vienna test system.

A modified version of the S12 Determination Task (DT, 32.00 version, Vienna Test Systems) was used to measure the speed of motor response, also called RT (Chong and Ong, 2015). This task was characterized by different temporal uncertainties of stimulus presentations. Specifically, the DT displayed 10 black-bordered white squares on a white background, arrayed in two horizontal rows of five. Each trial consisted of a square being temporarily filled with one of five different colors, namely, black, blue, green, yellow, or red, which appeared in one of 10 different locations (five in an upper row and five in a lower row). Participants were required to quickly press the corresponding colored button, using the dominant hand, to score a correct answer (see Figure 2 for an illustration of appearance terms).

Regardless of the speed of participant response, the colored square would remain constant for 1,250 ms before being superseded by the next trial, with a different random square now colored and another participant response required. The DT had a total duration of 950 s (including the familiarization and instruction phases). Familiarization phases were 300 s long and consisted of 20 stimuli. The instruction phase with a duration of 650 s consisted of three different trials with a total of 540 stimuli (79 white, 74 yellow, 78 red, 78 green, and 74 blue). Each trial had 180 stimuli with durations of 1,582, 948, and 1,078 ms for the first, second, and third trials, respectively. The same random sequence of stimulus presentation was used for all participants. Speed response as a measure of RT in the execution of the task (ms) was collected from each condition for final data analysis.

#### Cardiorespiratory Fitness

Cardiorespiratory fitness was assessed by an incremental treadmill cardiorespiratory exercise test using the athlete-led protocol (Draper and Marshall, 2014). Oxygen uptake was measured using a portable breath-by-breath expired air analyzer (K4b^2^Cosmed, Rome, Italy) weighing 1.5 kg. Data were transferred continuously via telemetry to a portable laptop. Breath-by-breath data were recorded continuously before, during, and after 5 min of running. Breath-by-breath data were averaged over 10-s intervals and exported to Excel and STATA for final data analysis. Heart rate and time to exhaustion were also collected.

### Statistical Analyses

All data were found to be normally distributed by Shapiro–Wilk and had equal variances. Participant characteristics are presented as mean and standard deviation (SD) for continuous variables and frequencies for categorical variables. Potential sex differences for each dependent variable were analyzed by *t*-test for continuous variables and chi-square tests for categorical variables. Pearson correlations were used to examine the relationship between attention tasks and descriptive climbing parameters. The Mallow Cp (Mallows, 1973) statistic regression model was used to find the optimum descriptive variables for the forecasting of attention tasks and climbing ability. Linear regression was performed to examine the association between climbing ability (on-sight or red-point) and attention task (AC or RT). In addition to the performance, covariates were included in the regression analyses. Specifically, three levels of adjustment were used: Model 1, unadjusted; Model 2, adjusted for sex, age, and climbing experience (years climbing); and Model 3, adjusted for sex, age, climbing experience (years climbing), and CRF (Cp Mallow: 3.38). Collinearity among the exposures was checked, and multicollinearity was not found in any of the models used. For all, the variance inflation factor was below 10, and the averaged variance inflation factor was close to 1 (Myers, 1990). It is important to highlight that two of the participants (both male) were excluded from just the RT (not AC) analyses because they were color blind. Statistical analyses were performed using STATA version 14.0 (Stata Corp, College Station, TX, United States). Statistical significance was set at *p* < 0.05.

## Results

Participant characteristics are shown in Table 1. Males were younger, heavier, and taller than females (*p* < 0.05). Moreover, males had a higher on-sight and red-point climbing ability with a greater CRF compared to females (*p* < 0.001 in all cases). Mean climbing experience was similar in both sexes.

Mean AC and RT measures in the attention tasks for all participants are presented in Table 2. No significant differences were found between male and female participants for any attention tasks, i.e., SIGNAL and DT.

**TABLE 2:** Mean (SD) attention tasks [accuracy of response (AC) and reaction time (RT)] for all participants and by sex.

	**All (*n* = 35)**	**Male (*n* = 25)**	**Female (*n* = 10)**
Accuracy of response (%)	87.6(6.1)	88.8(5.7)	84.8(6.1)
**Reaction time (ms)^a^**			
Trial 1	673.03 (50.59)	669.13 (56.4)	682 (34.58)
Trial 2	657.88 (48.07)	650.87 (49.99)	674 (41.15)
Trial 3	665.15 (46.65)	660 (46.71)	677 (46.68)
Total	665.8 (43.6)	660 (46.8)	679 (33.5)

*^*a*^Two daltonic participant were excluded from reaction time analyses, *n* = 33 (23 males).*

The relationship between AC and self-reported on-sight climbing ability is shown in Table 3. Full linear regression model analysis revealed that AC (measured by SIGNAL detection task), was positively related with the highest self-reported on-sight ability (β = 0.388; *p* = 0.031). However, there was no significant relationship between AC and self-reported red-point ability (β = 0.286; *p* = 0.064) (see Table 4).

**TABLE 3:** Relationship between accuracy of response (AC; dependent variable) and self-reported on-sight climbing ability (independent variable) in 35 experienced climbers.

	**β**	***p***	***R*^2^**	***R*^2^ adj**
Model 1	0.371	***0.028***	0.134	0.112
Model 2	0.278	*0.161*	0.191	0.083
Model 3	0.388	***0.031***	0.343	0.225

*Model 1: unadjusted; Model 2: sex, age, and climbing experience (years); Model 3: sex, age, climbing experience (years), and V̄O_2max_. β, beta, regression equation; LCI, lower confidence interval (95%); UCI, upper confidence interval (95%).*

**TABLE 4:** Relationship between accuracy of response (AC, dependent variable) and self-reported red-point climbing ability (independent variable) in 35 experienced climbers.

	**β**	***p***	***R*^2^**	***R*^2^ adj**
Model 1	0.308	*0.072*	0.095	0.067
Model 2	0.170	*0.182*	0.160	0.047
Model 3	0.286	*0.064*	0.298	0.172

*Model 1: unadjusted; Model 2: sex, age, and climbing experience (years); Model 3: sex, age, climbing experience (years), and V̄O_2max_. β, beta, regression equation.*

The relationship between RT and self-reported on-sight and red-point ability is presented in Tables 5, 6. Linear regression analysis revealed that there were no significant relationships between RT and self-reported on-sight (β = −0.102 to 0.020; *p* = 0.304 to 0.680) or red-point ability (β = −0.089 to 0.007; *p* = 0.306 to 0.893).

**TABLE 5:** Relationship between reaction time (RT; dependent variable) and self-reported on-sight climbing ability (independent variable) in 33 experienced climbers.

**RT**	**β**	***p***	***R*^2^**	***R*^2^ adj**
**Model 1**				
Trial 1	–0.086	*0.632*	0.008	–0.025
Trial 2	–0.075	*0.680*	0.006	–0.027
Trial 3	–0.102	*0.570*	0.011	–0.021
**Model 2**				
Trial 1	–0.033	*0.304*	0.154	0.033
Trial 2	0.015	*0.388*	0.133	0.009
Trial 3	–0.033	*0.519*	0.106	–0.022
**Model 3**				
Trial 1	–0.029	*0.442*	0.155	–0.002
Trial 2	0.020	*0.535*	0.134	–0.026
Trial 3	–0.024	*0.645*	0.111	–0.053

*Model 1: unadjusted; Model 2: sex, age, and climbing experience (years); Model 3: age, climbing experience (years), and VO_2max_; β, beta, regression equation; RT: reaction time.*

**TABLE 6:** Relationship between reaction time (RT; dependent variable) and self-reported red-point climbing ability (independent variable) in 33 experienced climbers.

	**β**	***p***	***R*^2^**	***R*^2^ adj**
**Model 1**				
Trial 1	–0.024	*0.893*	0.001	–0.032
Trial 2	–0.051	*0.780*	0.003	–0.030
Trial 3	–0.089	*0.621*	0.008	–0.024
**Model 2**				
Trial 1	–0.017	*0.306*	0.153	0.032
Trial 2	0.007	*0.389*	0.133	0.009
Trial 3	–0.066	*0.506*	0.108	–0.019
**Model 3**				
Trial 1	–0.010	*0.444*	0.155	–0.003
Trial 2	0.014	*0.536*	0.134	–0.026
Trial 3	–0.052	*0.637*	*0.113*	–0.051

*Model 1: unadjusted; Model 2: sex, age, and climbing experience (years); Model 3: sex, age, climbing experience (years), and VO_2max_; β, beta, regression equation.*

## Discussion

The current study is the first to assess the relationship between attention and self-reported climbing ability (on-sight and red-point) in rock climbers. Further, it is the first to assess how potential confounding factors may affect the predictive attention functioning. The results suggested that attention is significantly related to on-sight but not red-point climbing ability.

Greater levels of attention in higher-ability climbers suggest two possibilities. First, there is a degree of self-selection, with the higher-ability climbers’ performance occurring because of naturally greater levels of attention. Second, and more likely, is that higher-ability climbers develop better attention, through repeated practice of the climbing task that requires them to detect the hand and footholds when they climb on-sight, as suggested by the “cognitive abilities hypothesis.” This hypothesis focuses on the direct relationship between sport practice and the cognitive abilities which could be associated with the interaction between the athlete and their specialized environment (Singer, 2000; Mann et al., 2007). The hypothesis suggests that if volitional and repeated practice is the driving force behind mechanisms of brain plasticity (Debarnot et al., 2014), then sport type may be also a potential moderator of the sport–cognition relationship (Fetz, 2007; Voss et al., 2010). As such, different sports appear to have different influences on cognitive functioning. For instance, Lum et al. (2002) observed that athletes from externally paced sports (i.e., soccer) were better at voluntarily orienting attention to locations where useful information was, whereas athletes from self-paced sports (i.e., swimming) and/or nonathletes were not as good at voluntary orienting attention. This may in part explain why dynamic sports place a high premium on voluntary allocation of spatial attention. Kioumourtzoglou et al. (1998) analyzed perceptual speed, prediction, selective attention, decision making, focused attention, estimation of speed and direction of a moving object, visual RT, and spatial orientation between experts and novice basketball, volleyball, and water polo players. The authors found that expert basketball players had better selective attention compared to novices. In addition, expert volleyball players had better focused attention, prediction, and estimation of speed and direction of a moving object compared to novices. Lastly, water polo players had significantly better decision making, visual RT, and spatial orientation than novices. As such, in the current study, the “cognitive abilities hypothesis” may in part explain why a better attention in higher-level climbers could be associated with the characteristics of a difficult route (i.e., small holds are more difficult to find).

Analyses, shown in Tables 5, 6, revealed that there was no relationship between RT and either on-sight or red-point ability. The absence of a relationship between RT and ability, together with the differences found in AC, is consistent with the findings of Wang et al. (2013). Here, the authors suggest that differences in the execution of tasks and attention may be found depending on the nature of the sport, i.e., whether it is self-paced or externally paced. The absence of differences in RT measures between ability groups could indicate the type of perceptual–cognitive abilities required when climbing. The self-paced nature of climbing means that the athletes’ performance is less influenced by temporal pressure and more by the AC. However, it is also possible that an expert performance would be characterized by a more strategic and adapted allocation of the attentional resources (Bourdin et al., 1998). Future research should investigate this using a sample encompassing athletes with a range of different abilities from a variety of sports. This would help to clarify the potential differences between ability levels and types of sport (self-paced vs. externally paced).

Previous research investigating the relationship between fitness and performance in attention tasks (Luque-Casado et al., 2016; Ciria et al., 2017) suggested that CRF was an important mediator. However, our results do not support such a relationship. This divergent finding may be because previous research used different attention tasks and has compared sedentary participants with trained athletes, whereas the present study used only trained athletes albeit with different ability levels. In addition, the importance of CRF and attention has been investigated in sports where CRF is the primary factor for performance, such as triathlon and cycling. However, our data provide evidence in favor of the “hypothesis of cognitive abilities” associated with the interaction between the athlete and their specialized environment (Singer, 2000).

Climbing is a sport that demands attention to progress along the path without falls or failures and without attending to factors external to the task (e.g., risk to fall) which could affect performance. These performance advantages in AC on the attention task could explain why better climbers are less affected by anxiety when on-sight climbing, as previously reported by Draper et al. (2011b), particularly given that anxiety results in part from the failure of the attention network (Ghassemzadeh et al., 2019). As such, the stronger relationship between attention and on-sight climbing ability could also be explained by the absence of anxiety seen in advanced climbers (Fryer et al., 2013), and thus, they may have an enhanced ability to focus on the physical movements. One possible explanation for this difference in the AC in the attention task could be the general training of climbers, since during climbing, long periods of attention (i.e., monitoring) are required for good performance (Bourdin et al., 1998). As suggested by Pijpers et al. (2006), emotional state (anxiety) affects the attentional control and realization of affordances, but the inverse may be also possible. In this sense, we hypothesized that climbers with better attention would be less affected by the negative effect of anxiety, by focusing only on the relevant aspects of a climbing task such as the hold type or foot placement.

The current study did not reveal any relationships between attention and red-point climbing ability. This finding is likely explained by the different characteristics between red-point and on-sight types of climbing. While on-sight climbing requires visual inspection of the route to look for the best/next hold to keep ascending, a red-point ascent is defined by a climber’s previous knowledge of the route, its holds, and the movement sequences required; thus, the attentional demands of the red-point style are likely lower. The current study supports the idea that on-sight climbing requires greater attentional demand compared to red-point climbing, and this may contribute to the development of better attention. This is an important finding that may indicate that the on-sight climbing style could have a considerable positive learning effect on attention, which could be an important component of competitive climbing performance. However, further research is needed to assess if this learning effect exists and whether it can be trained. As such, coaches and trainers should consider including strategies based on practicing on-sight climbing styles instead of the red-point climbing style.

The findings of the current study may help explain why better climbers are less affected by anxiety during an on-sight ascent as seen in previous studies (e.g., Draper et al., 2011b). Further, greater attention accuracy in better climbers could imply a better climbing efficiency or perceptual motor performance (efficient exploration and decrease in the number of typical exploratory movements) (Orth et al., 2017). This could be very important for competitive climbing, given that international competitions use on-sight climbing and a grade of 0.4 (IRCRA) separated the top four competitors in the 2015 International Federation Sport Climbing World Cup (Fryer et al., 2016). We have reported a larger association than a grade between attention and on-sight climbing ability. This is another key finding that could suggest that attention or attentional demands may be important aspects of competitive rock climbing performance. However, the small *R*^2^ (0.14 and 0.32) still supports the concept that climbing performance is a multifactorial sport. As such, there are likely to be other cognitive skills (working memory, inhibitory control, cognitive flexibility, reasoning, etc.) or attentional functions (alert, orienting, or executive control) (Roca et al., 2018) that might explain large percentages of variance that we have not measured in the current study. However, our finding is still important given that at the top level in climbing, marginal gains are key to success.

While this research has presented important and unique findings, to fully contextualize the data, several limitations should be acknowledged. We performed power analyses on the multiple regression models presented in this study. For a level of significance of 0.05, with the current sample size, taking into account the *R*^2^ of all the covariates in the model and the number of covariates, we observed a power of 64.9–84.8% for the main models. The experimental design allows us to reveal how the variables are related, but it does not allow us to detect possible cause–effect relationships. In addition, this study does not allow us to determine whether there are any differences between climbers and non-climbers. Given these points, future research should (1) increase the sample size, (2) conduct an intervention to confirm whether improving the ability of a climber actually improves execution in attention or even knowing whether climber with better attention is less affected by anxiety, and finally (3) study possible differences in attention as a consequence of climbing training between climbers and non-climbers.

## Conclusion

In summary, our results suggest that after controlling for potential confounding factors (sex, age, climbing experience, and CRF), attention measured objectively is positively related to on-sight, but not red-point, climbing ability. This may be explained by the different ascent characteristics; in particular, the greater attentional demands of on-sight lead climbing may be due to the lack of information regarding the route. The lack of association between RT and climbing performance may be due to the self-paced nature of the sport, as little external temporal demand is placed on the athlete. Rock climbers and their coaches should consider attentional training for on-sight climbing performance in order to increase or maintain climbing ability.

## Data Availability Statement

All datasets generated for this study are included in the article/supplementary material.

## Ethics Statement

The studies involving human participants were reviewed and approved by Comité de Ética para la Experimentación Biomédica y de Evaluación de Experimentación con Organismos Modificados Genéticamente (CEED/OMGs) from Cadiz University. The patients/participants provided their written informed consent to participate in this study.

## Author Contributions

IG-P wrote the first draft of the manuscript. VE-R performed the statistical analyses and together to SF and DG conceived and designed the study and contributed to manuscript development and writing until manuscript was submitted in its final version. JG-R has contributed to the reading and suggestions of the final version.

## Conflict of Interest

DG was employed by Lattice Training Ltd. while working on this manuscript.

The remaining authors declare that the research was conducted in the absence of any commercial or financial relationships that could be construed as a potential conflict of interest.
